# A novel scoring model for predicting prolonged mechanical ventilation in cardiac surgery patients: development and validation

**DOI:** 10.3389/fcvm.2025.1573874

**Published:** 2025-03-26

**Authors:** Quan Liu, Pengfei Chen, Wuwei Wang, Yifei Zhou, Yichen Xu, Xu Cao, Rui Fan, Wen Chen, Fuhua Huang, Xin Chen

**Affiliations:** ^1^ School of Medicine, Southeast University, Nanjing, Jiangsu, China; ^2^ Department of Thoracic and Cardiovascular Surgery, Nanjing First Hospital, Nanjing Medical University, Nanjing, Jiangsu, China; ^3^ Cardiovascular Surgery Department, Fuwai Hospital, Chinese Academy of Medical Sciences and Peking Union Medical College, National Center for Cardiovascular Diseases, Beijing, China

**Keywords:** prolonged mechanical ventilation, cardiac surgery, predicting model, multiple centers, retrospective study

## Abstract

**Objective:**

Prolonged mechanical ventilation (PMV) is a significant postoperative complication in cardiac surgery, associated with increased mortality and healthcare costs. This study aims to develop and validate a novel scoring model to predict the risk of PMV in cardiac surgery patients.

**Methods:**

A retrospective analysis was conducted using data from 14 comprehensive hospitals in Jiangsu Province, including adult patients who underwent coronary artery bypass grafting (CABG), valve surgery, and aortic surgery from January 2021 to December 2022. Predictive variables were selected based on clinical expertise and prior literature, and a nomogram was developed using LASSO regression and multiple logistic regression. Model performance was evaluated using the C-index, calibration plots, and decision curve analysis (DCA).

**Results:**

A total of 5,206 patients were included in the final analysis. The incidence rate of PMV were 11.83% in the training set, 8.65% in the internal validation set, and 15.4% in the external validation set. The nomogram identified 9 significant predictors, including age, gender, preoperative conditions, and surgical factors. The model demonstrated robust performance with C-index values of 0.79 in the training and internal validation sets and 0.75 in the external validation set, indicating good predictive capability. Calibration curves confirmed the accuracy of predicted probabilities, and DCA indicated substantial net benefits for clinical decision-making.

**Conclusions:**

This study presents a validated scoring model for predicting PMV in cardiac surgery patients, integrating a comprehensive range of clinical variables. The model facilitates early identification of high-risk patients, enabling tailored perioperative strategies and potentially improving patient outcomes and resource utilization in cardiac surgery.

## Introduction

Despite advancements in the perioperative management of cardiac surgery patients, prolonged mechanical ventilation (PMV) remains a significant postoperative challenge, with reported incidence rate reaching as high as one in five patients (1–3). PMV is associated with increased mortality, reduced quality of life, and substantial economic burden (4–6). Several recent studies and the Society of Thoracic Surgeons (STS) identify PMV lasting more than 24 h as a major morbidity endpoint in cardiac surgery, which aligns with our study's definition of PMV as a duration exceeding 24 h on the ventilator (1, 7, 8).

PMV following cardiac surgery places a considerable burden on both patients and healthcare systems, underscoring the need for a reliable predictive model to facilitate early detection and management. While previous studies have identified predictive factors for PMV after cardiac surgery, these efforts have often been limited by small sample sizes, population homogeneity, and the absence of independent external validation cohorts (2, 9, 10). To address these limitations, we conducted a retrospective analysis using data from 14 hospitals to develop a predictive model for the risk of PMV after cardiac surgery, which we subsequently validated in an independent cohort.

## Methods

### Patient selection

This study was a retrospective, observational research project that included inpatient records of adult patients who underwent CABG/valve surgery and aortic surgery, including combined procedures, at 14 level-three grade A comprehensive hospitals in Jiangsu Province from January 2021 to December 2022. Patients aged under 18 years, those who required preoperative intubation, had preoperative circulatory instability, were critically ill, underwent cardiac transplantation, received left ventricular assist devices, or underwent other non-cardiac open surgeries were excluded from the study. PMV was defined as the requirement for mechanical ventilation for more than 24 h following cardiac surgery. The research was approved by the Ethical Committee of Nanjing First Hospital (KY20170811-03), and patient informed consent was waived due to the retrospective nature of the study.

### Variables selection

The selection of predictive variables in this study was based on clinical expertise and prior findings reported in the existing literature. Initially, demographic data including age, gender, height, weight, and smoking history were collected. Additionally, preoperative status and certain biomarkers were included in the dataset. These included diabetes, insulin use, preoperative hypertension, preoperative hyperlipidemia, preoperative dialysis, peripheral artery disease, chronic lung disease, history of cerebrovascular accidents, atrial fibrillation, history of percutaneous coronary intervention (PCI), preoperative serum creatinine, preoperative total bilirubin, preoperative hemoglobin, preoperative left ventricular ejection fraction (LVEF), and whether there was significant left main coronary artery disease. Moreover, surgical-related variables were collected, including whether the surgery was an emergency, the use of cardiopulmonary bypass, the duration of cardiopulmonary bypass, intraoperative red blood cell transfusion, intraoperative plasma transfusion, intraoperative cryoprecipitate transfusion, coronary artery bypass grafting, valve surgery, and aortic surgery.

### Statistical analysis

Patients from Nanjing First Hospital were randomly divided into a training set and an internal validation set in a 7:3 ratio, while patients from other cardiac centers served as an external validation set. Continuous variables were presented as mean ± standard deviation. Student's *t*-test was employed to compare differences between groups. Categorical variables were reported as frequencies and percentages, and the Rao-Scott chi-square test was used for comparisons.

The variables in the training set underwent a filtering process via LASSO regression. After obtaining the predictors through LASSO regression, we constructed prediction models based on the multivariate logistic regression. Variables with non-zero coefficients in the LASSO regression model were selected to develop the nomogram prediction model. The accuracy of the risk prediction model was evaluated using several metrics, including the receiver operating characteristic curve (ROC) curve, calibration plot, and decision curve analysis (DCA) curve. These evaluations were performed for the training set, internal validation set, and external validation set. An area under the curve (AUC) value closer to 1 indicates better predictive capability of the model. Moreover, an AUC value greater than 0.7 signifies that the model has good predictive capacity. The calibration plot displays a scatter plot of the observed vs. predicted incidence; if the curves lie along the diagonal of the coordinate system, it indicates greater accuracy in the model's predictive ability. The DCA curve circumvents the issues associated with selecting cut-off values for the ROC curve, sensitivity, and specificity, directly calculating the net benefit in clinical settings. A DCA curve that remains above the two extremes suggests good clinical applicability of the model. In all the analyses mentioned above, a two-tailed *p*-value less than 0.05 was considered statistically significant.

## Results

### Characteristics of the cohorts

**Figure 1:**
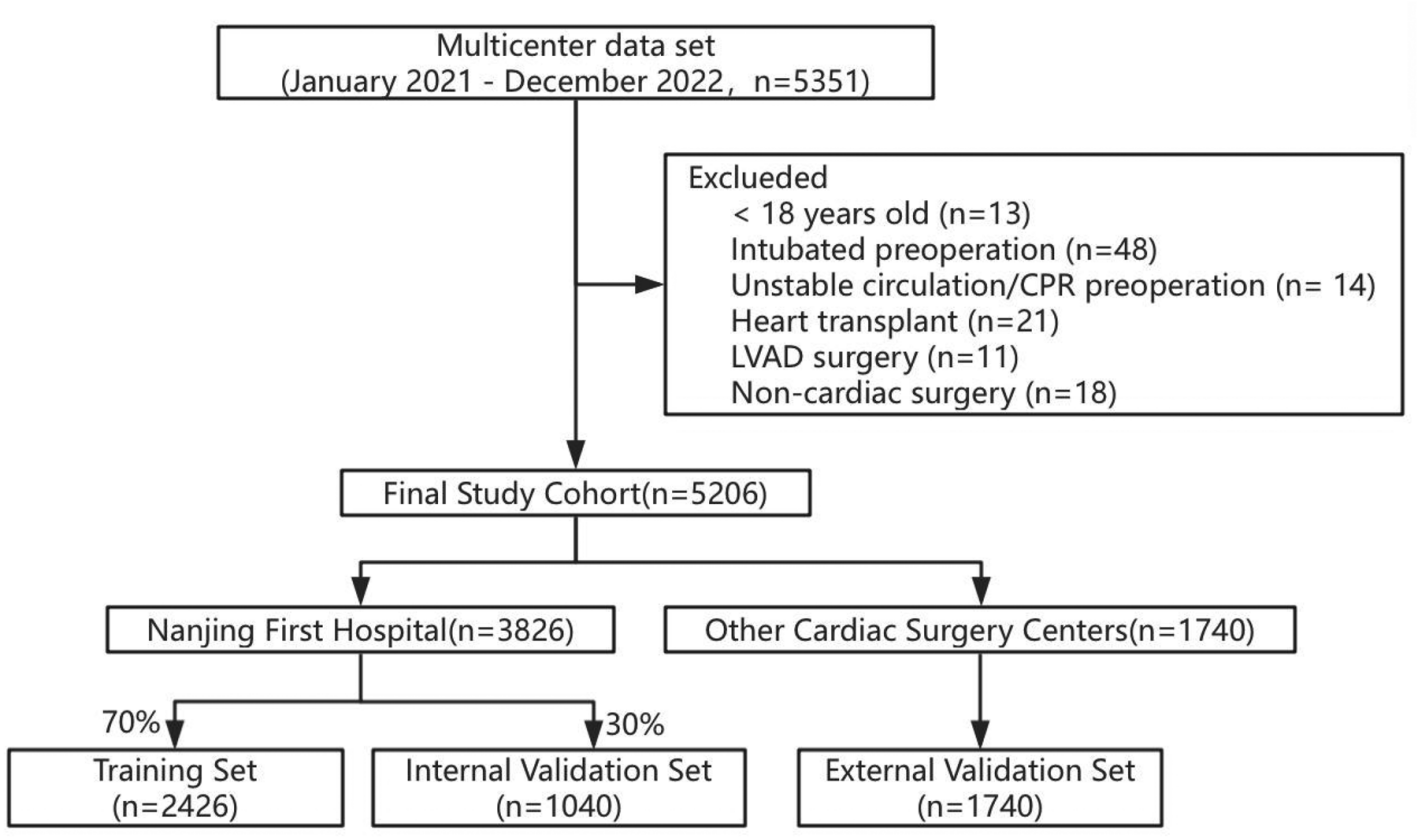
Participant selection and dataset division.

During the period spanning from January 2021 to December 2022, a comprehensive analysis was conducted involving a total of 5,351 participants. We excluded 13 individuals under the age of 18, 48 individuals who were intubated preoperatively, 14 individuals with unstable circulation or who underwent cardiopulmonary resuscitation before surgery, 21 individuals who had heart transplants, 11 individuals who underwent left ventricular assist device (LVAD) surgery, and 18 individuals who had non-cardiac valve surgeries, resulting in a final study cohort of 5,206 individuals (Figure 1).

Among them, 3,826 individuals were from the patient cohort of Nanjing First Hospital, and the remaining 1,740 individuals were from 13 other cardiac centers. Using the random seed method, the patient queue of Nanjing First Hospital was divided into a training set (*N* = 2,426) and an internal validation set (*n* = 1,040) in a ratio of 7:3. The patient queues of the other 13 cardiac centers were used as an external validation set (*n* = 1,740) (Figure 1).

Baseline characteristics differed significantly across the 3 groups. The incidence rate of PMV in the training set, internal validation set, and external validation set were 11.83%, 8.65%, and 15.4% respectively (Table 1). In the training set, patient characteristics of the PMV and non-PMV groups were compared (Table 2).

**Table 1:** Clinical characteristics in training set, internal and external validation set.

Characteristics	Training set (*N* = 2,426)	Internal validation set (*N* = 1,040)	External validation set (*N* = 1,740)	*P* value
Age	61.47 ± 11.6	61.44 ± 11.54	62.86 ± 10.42	<0.001
Sex				<0.001
Male	1,495 (61.62)	639 (61.44)	1,318 (75.75)	
Female	931 (38.38)	401 (38.56)	422 (24.25)	
BMI	24.25 ± 3.44	24.21 ± 3.31	24.54 ± 3.29	0.010
Medical history				
Smoking	853 (35.16)	364 (35)	703 (40.4)	<0.001
Diabetes	598 (24.65)	241 (23.17)	553 (31.78)	<0.001
Insulin-treated diabetes	252 (10.39)	88 (8.46)	209 (12.01)	0.012
Hypertension	1,371 (56.51)	552 (53.08)	1,203 (69.14)	<0.001
Hyperlipidemia	261 (10.76)	105 (10.1)	359 (20.63)	<0.001
Dialysis	19 (0.78)	7 (0.67)	18 (1.03)	0.543
Peripheral arterial disease	50 (2.06)	21 (2.02)	32 (1.84)	0.874
Chronic lung disease	60 (2.47)	34 (3.27)	44 (2.53)	0.379
Carotid artery stenosis				<0.001
None	2,312 (95.3)	996 (95.77)	1,460 (83.91)	
Unilateral	69 (2.84)	27 (2.6)	83 (4.77)	
Bilateral	45 (1.85)	17 (1.63)	197 (11.32)	
Previous cerebrovascular accident	224 (9.23)	84 (8.08)	164 (9.43)	0.452
Atrial fibrillation	470 (19.37)	205 (19.71)	42 (2.41)	<0.001
Previous PCI	155 (6.39)	56 (5.38)	128 (7.36)	0.118
LVEF	62 (57, 64)	62 (58, 64)	61 (54, 65)	0.253
Left main disease	275 (11.34)	114 (10.96)	531 (30.52)	<0.001
Laboratory test				
Creatinine	73.95 (62, 88.84)	72 (61.58, 86.78)	76 (63, 91)	<0.001
Bilirubin	10.7 (7.7, 15.32)	10.8 (7.8, 15.5)	11.3 (8.3, 16.1)	0.003
Hemoglobin	132 (120, 143)	132 (121, 143)	129 (118, 141)	<0.001
Surgical information				
Emergency	170 (7.01)	79 (7.6)	176 (10.3)	<0.001
CPB	2,287 (94.27)	982 (94.42)	1,599 (91.9)	0.004
CPB time	112 (85, 144)	114 (84, 144.25)	115 (81, 145)	0.906
Intraoperative RBC transfusion	246 (10.14)	125 (12.02)	679 (39.02)	<0.001
Intraoperative plasma transfusion	138 (5.69)	70 (6.73)	530 (30.46)	<0.001
Intraoperative cryoprecipitate transfusion	439 (18.1)	183 (17.6)	337 (19.37)	0.432
CABG	1,108 (45.67)	448 (43.08)	1,445 (83.05)	<0.001
Valve surgery	1,396 (57.54)	621 (59.71)	266 (15.29)	<0.001
Aorta surgery	403 (16.61)	184 (17.69)	284 (16.32)	0.630
In-hospital outcomes				
Postoperative reintubation	64 (2.64)	31 (2.98)	40 (2.3)	0.539
In-hospital death	64 (2.64)	27 (2.6)	35 (2.01)	0.396
PMV	287 (11.83)	90 (8.65)	268 (15.4)	<0.001

Values are presented as mean ± SD, median (quartiles) or *n* (%).

BMI, body mass index; PCI, percutaneous coronary intervention; LVEF, left ventricular ejection function; CPB, cardiopulmonary bypass; RBC, red blood cell; CABG, coronary artery bypass grafting; PMV, prolonged mechanical ventilation.

**Table 2:** Characteristics of patients with PMV and non-PMV in training set.

Characteristics	Non-PMV (*N* = 2,139)	PMV (*N* = 287)	*P* value
Age	61.28 ± 11.60	62.92 ± 11.54	0.024
Sex			0.760
Male	1,321 (61.76)	174 (60.63)	
Female	818 (38.24)	113 (39.37)	
BMI	24.22 ± 3.37	24.50 ± 3.90	0.239
Medical history			
Smoking	749 (35.02)	104 (36.24)	0.733
Diabetes	531 (24.82)	67 (23.34)	0.636
Insulin-treated diabetes	225 (10.52)	27 (9.41)	0.634
Hypertension	1,178 (55.07)	193 (67.25)	<0.001
Hyperlipidemia	236 (11.03)	25 (8.71)	0.275
Dialysis	16 (0.75)	3 (1.05)	0.485
Peripheral arterial disease	39 (1.82)	11 (3.83)	0.042
Chronic lung disease	45 (2.1)	15 (5.23)	0.003
Carotid artery stenosis			0.109
None	2,041 (95.42)	271 (94.43)	
Unilateral	56 (2.62)	13 (4.53)	
Bilateral	42 (1.96)	3 (1.05)	
Previous cerebrovascular accident	196 (9.16)	28 (9.76)	0.828
Atrial fibrillation	404 (18.89)	66 (23)	0.115
Previous PCI	134 (6.26)	21 (7.32)	0.578
LVEF	62 (57, 64)	61 (49, 64)	<0.001
Left main disease	247 (11.55)	28 (9.76)	0.424
Laboratory test			
Creatinine	73 (62, 87.75)	79.8 (64, 105.5)	<0.001
Bilirubin	10.6 (7.6, 14.9)	12.1 (8.25, 18.2)	<0.001
Hemoglobin	132 (120, 143)	129 (118, 141)	0.027
Surgical information			
Emergency	90 (4.21)	80 (27.87)	<0.001
CPB	2,005 (93.74)	282 (98.26)	0.003
CPB time	108 (83, 137)	147 (111.5, 177.5)	<0.001
Intraoperative RBC transfusion	189 (8.84)	57 (19.86)	<0.001
Intraoperative plasma transfusion	87 (4.07)	51 (17.77)	<0.001
Intraoperative cryoprecipitate transfusion	311 (14.54)	128 (44.60)	<0.001
CABG	992 (46.38)	116 (40.42)	0.066
Valve surgery	1,220 (57.04)	176 (61.32)	0.188
Aorta surgery	308 (14.40)	95 (33.10)	<0.001
In-hospital outcomes			
Postoperative reintubation	15 (0.7)	49 (17.07)	<0.001
In-hospital death	26 (1.22)	38 (13.24)	<0.001

Values are presented as mean ± SD, median(quartiles) or *n* (%).

PMV, prolonged mechanical ventilation; BMI, body mass index; PCI, percutaneous coronary intervention; LVEF, left ventricular ejection function; CPB, cardiopulmonary bypass; RBC, red blood cell; CABG, coronary artery bypass grafting.

### Feature selection and nomogram

**Figure 2:**
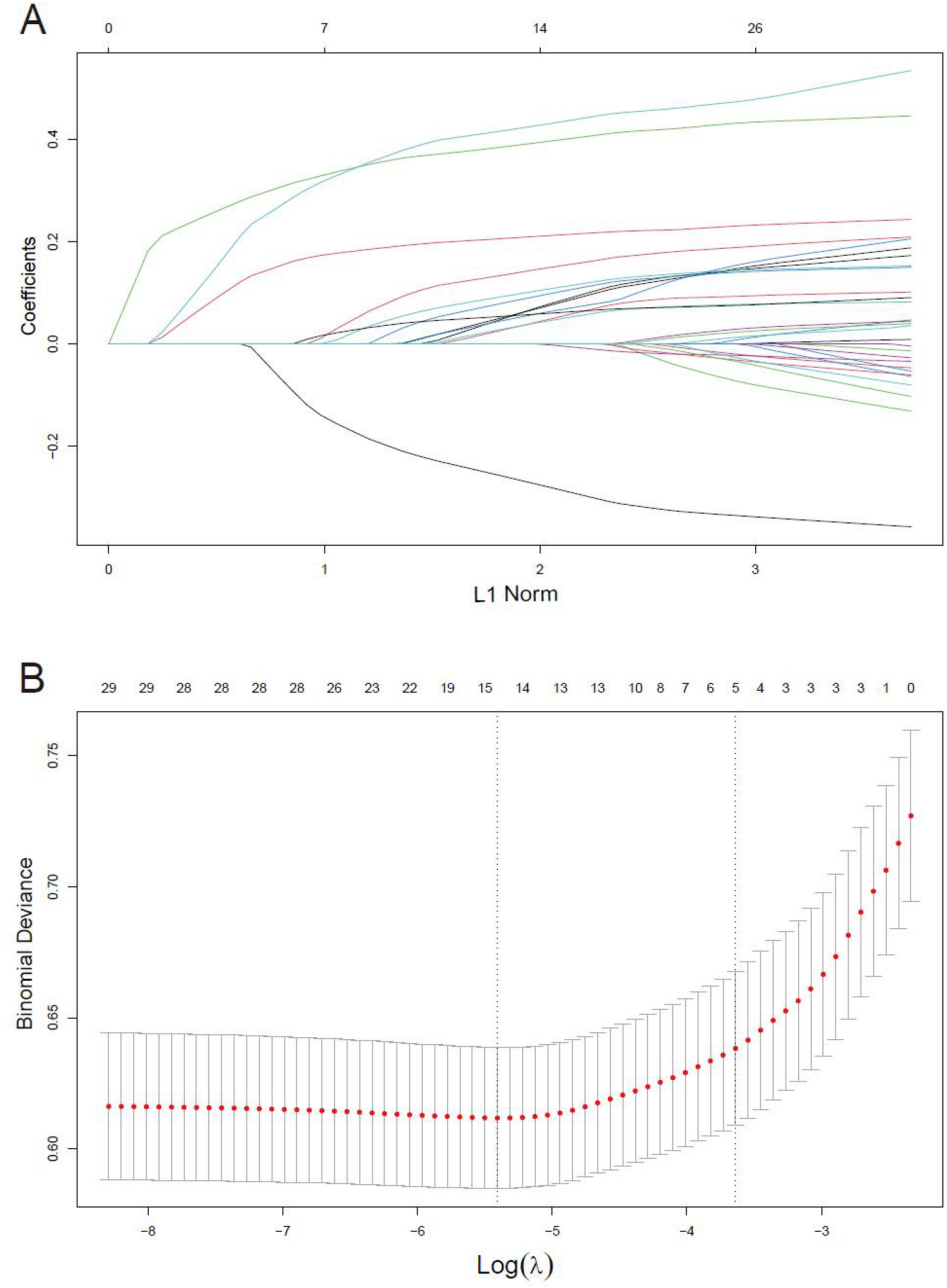
Variables selection using the LASSO regression models. **(A)** LASSO coefficient profiles of the 29 variables. Variables are included in the LASSO regression model for selection, the regression coefficients progressively diminish towards zero with increasing penalty. **(B)** Optimal parameter (lambda) selection in the LASSO model used 10-fold cross-validation via minimum criteria.

When 29 variables are included in the LASSO regression model for variable selection, the regression coefficients of all variables progressively diminish towards zero with increasing penalty, ultimately converging to zero (Figure 2A). The significant variables were determined through ten-fold cross-validation, illustrated in Figure 2B. Ultimately, 14 variables were identified, encompassing gender, age, preoperative condition, hypertension, peripheral arterial disease, chronic lung disease, preoperative atrial fibrillation, blood creatinine level, preoperative bilirubin level, preoperative hemoglobin level, LVEF value, emergency surgery status, cardiopulmonary bypass duration, intraoperative plasma transfusion, and intraoperative cryoprecipitate transfusion.

**Figure 3:**
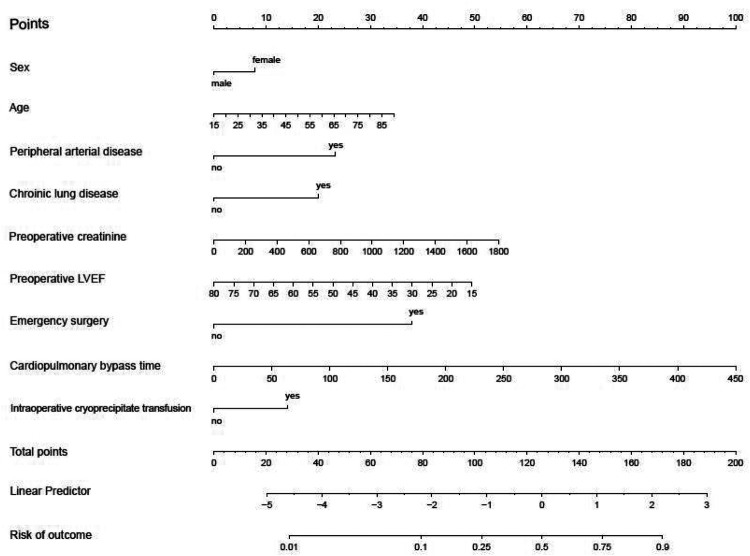
Nomogram derived from training set for predicting prolonged mechanical ventilation.

Subsequently, these 14 variables were incorporated into a multiple logistic regression model (Table 3). The analysis unveiled that gender, age, peripheral arterial disease, chronic lung disease, preoperative creatinine level, LVEF value, emergency surgery status, cardiopulmonary bypass duration, and intraoperative cryoprecipitate transfusion emerged as independent risk factors for postoperative PMV in cardiac surgery patients. A nomogram model was developed utilizing these 9 variables (Figure 3).

**Table 3:** Multivariate logistic regression of predictors associated with prolonged mechanical ventilation in the training set.

Variables	OR	95%CI	*P*-value
Sex			0.026
Male	Ref	–	
Female	1.42	(1.04, 1.93)	
Age	1.02	(1.00, 1.03)	0.012
Hypertension	1.38	(1.03, 1.87)	0.033
Peripheral arterial disease	3.00	(1.39, 6.50)	0.005
Chronic lung disease	2.74	(1.41, 5.32)	0.003
Atrial fibrillation	1.37	(0.97, 1.94)	0.066*
Preoperative creatinine	1.00	(1.00, 1.00)	0.046
Preoperative bilirubin	1.01	(1.00, 1.02)	0.092*
Preoperative hemoglobin	1.01	(0.99, 1.01)	0.656*
Preoperative LVEF	0.96	(0.95, 0.98)	<0.001
Emergency surgery	5.61	(3.59, 8.76)	<0.001
Cardiopulmonary bypass time	1.01	(1.01, 1.01)	<0.001
Intraoperative plasma transfusion	1.43	(0.89, 2.30)	0.145*
Intraoperative cryoprecipitate transfusion	1.83	(1.29, 2.59)	<0.001

OR, odds ratio; CI, confidence interval; LVEF, left ventricular ejection function.

* *P* > 0.05.

### Model performance

**Figure 4:**
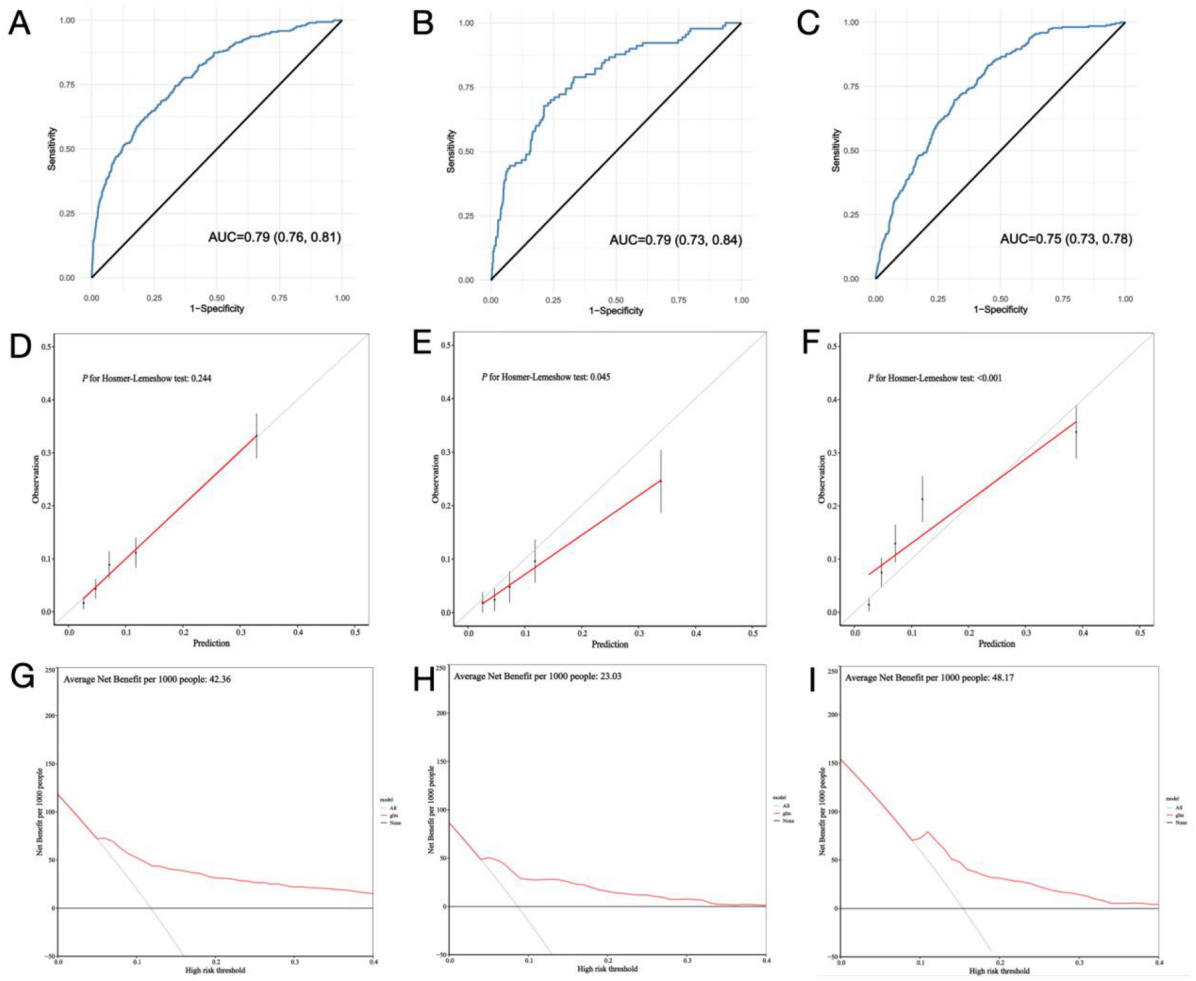
Model performance of the nomogram for predicting prolonged mechanical ventilation after cardiac surgery in train set **(A,D,G)**, internal validation set **(B,E,H**) and external validation set **(C,F,I)**.

To assess the robustness of the clinical prediction model established, we conducted testing on the training set, internal validation set, and external validation dataset. Initially, we computed the model's C-index. In the training set, the nomogram model's C-index was 0.79 (95% confidence interval, 0.76–0.81; *p* < 0.001), while in the internal and external validation sets, the C-index was 0.79 (95% confidence interval, 0.73–0.84; *p* < 0.001) and 0.75 (95% confidence interval, 0.73–0.78; *p* < 0.001), respectively. Calibration curves indicated that the fitted actual occurrence rate of PMV (*Y*-axis) and the predicted occurrence rate (*X*-axis) in the nomogram model were distributed around a line with a slope of approximately 45°. The decision curve, within the horizontal coordinate range of 0.1–0.4, positioned above the lines representing None and All, suggesting that the model exhibits good predictive capability within this range (Figure 4).

## Discussion

PMV is a significant postoperative complication in cardiac surgery, impacting patient outcomes and healthcare resources (11). Our study corroborates that inpatient mortality rate is higher in the PMV group compared to the non-PMV group. While most studies use a 24 or 48 h threshold for extubation time to determine PMV duration, our research aligns with previous findings by adopting a 24-h threshold, given its association with faster recovery post-surgery (1, 12, 13).

This study aimed to address the challenge of predicting PMV by developing and validating a novel scoring model. The nomogram was constructed based on a rigorous selection process that identified nine key predictors, chosen for their statistical significance and clinical relevance. This model incorporates a comprehensive range of factors, including patient demographics, comorbidities, and intraoperative variables, reflecting the multifactorial nature of PMV. Its primary strength lies in using multi-center data, which encompasses a large sample size and includes an independent external validation cohort.

Age and chronic lung disease are common risk factors for PMV after cardiac surgery (13–16). The female gender was found to be independently associated with failure of the spontaneous breathing trial and failure of prolonged weaning in a previous study (2). The main reason for this may be related to the changes in female hormone levels. Sharma et al. reveals that lower LVEF is a significant predictor of PMV following cardiac surgery (12). Huan et al. identified LVEF as a critical predictor influencing the occurrence of PMV after robot-assisted CABG (14). Patients with a low ejection fraction may experience greater fluctuations in circulation after cardiac surgery. Since stable circulation is a prerequisite for extubation, these patients are likely to require prolonged mechanical ventilation. Many studies have confirmed that cardiopulmonary bypass (CPB) time are independent risk factors for PMV (9, 12, 14, 17, 18). Prolonged CPB can trigger a systemic inflammatory response that activates various mediators, and disrupt normal physiological homeostasis, leading to increased capillary permeability in the pulmonary circulation (12).

Due to systemic atherosclerosis, patients with peripheral arterial disease may also experience indirect effects on their lungs, leading to reduced pulmonary function (19). After surgery, these patients may require prolonged mechanical ventilation to maintain adequate oxygenation levels. Patients with elevated preoperative creatinine levels often face more complex fluid management issues postoperatively after cardiac surgery (7, 20–22). If fluid management is not handled properly, it may lead to pulmonary edema or other respiratory-related complications, which can delay weaning from mechanical ventilation. Patients undergoing emergency surgery are often in a severe state, potentially experiencing shock, severe infections, or acute heart failure (9). These factors increase the risk of surgery and may result in delayed postoperative recovery of respiratory function, necessitating a longer duration of mechanical ventilation. The intraoperative administration of large amounts of blood products, such as cryoprecipitate, can trigger a systemic inflammatory response (23–26). This response may affect lung function, leading to delayed recovery of pulmonary function postoperatively, thereby prolonging the duration of mechanical ventilation.

To illustrate the model's application, consider a 50-year-old female patient with peripheral vascular disease and chronic pulmonary disease, a preoperative serum creatinine level of 200 µmol/L, and an LVEF of 50%. She underwent emergency cardiac surgery with a 100-min CPB time and received cryoprecipitate. The patient would receive 8 points for female, 16 points for age, 23 for peripheral vascular disease, 20 points for chronic pulmonary disease, 6 points for serum creatinine, 23 points for LVEF, 38 points for emergency surgery, 22 points for CPB and 14 points for cryoprecipitate for a score of 170, indicating a 90% risk of PMV.

Hypertension was initially included in model construction but was subsequently excluded due to its limited predictive value for PMV and adverse effects on model performance. The model demonstrated strong performance across various datasets, with consistent C-index values of 0.79 in both the training and internal validation sets and 0.75 in the external validation set, reflecting high accuracy in distinguishing between high- and low-risk patients for PMV. Heng Yang et al. developed a potential nomogram to predict the risk of PMV after valve surgery in a single-center retrospective study with a C-index of 0.782 (27), which indicate the robustness of our model. Calibration curves confirmed that predicted probabilities closely matched actual outcomes. The decision curve analysis revealed substantial net benefits in clinical decision-making within a probability threshold range of 0.1–0.4, emphasizing the model's practical value in guiding early interventions and resource allocation.

This predictive model has significant implications. It allows for early identification of high-risk patients, enabling more tailored perioperative strategies, such as increased surveillance, proactive respiratory support, and optimized comorbidity management. Additionally, the model can facilitate informed discussions with patients and families about expected postoperative courses and potential interventions. It also offers potential benefits in resource utilization, reducing ICU stays, ventilator dependency, and associated healthcare costs.

Despite promising results, this study has limitations. The retrospective nature of the data may introduce biases, and variability in clinical practices across multiple centers could affect the generalizability of the findings. For example, NT-ProBNP, NYHA class was excluded due to grossly incomplete data. Although the model performed well in the external validation cohort, further prospective studies are needed to validate its effectiveness in broader and more diverse patient populations. Future research should explore incorporating emerging biomarkers and integrating machine learning techniques to enhance predictive accuracy (28).

## Conclusion

Our study introduces a validated scoring model for predicting PMV in cardiac surgery patients. By incorporating a range of clinical variables, the model provides a practical tool for improving perioperative planning and patient care. Its adoption could enhance outcomes through early identification and management of at-risk patients, advancing the field of cardiac surgery and postoperative care.

## Data Availability

The raw data supporting the conclusions of this article will be made available by the authors, without undue reservation.
